# Origin of the São Paulo Yellow Fever epidemic of 2017–2018 revealed through molecular epidemiological analysis of fatal cases

**DOI:** 10.1038/s41598-019-56650-1

**Published:** 2019-12-31

**Authors:** Marielton dos Passos Cunha, Amaro Nunes Duarte-Neto, Shahab Zaki Pour, Ayda Susana Ortiz-Baez, Jiří Černý, Bárbara Brito de Souza Pereira, Carla Torres Braconi, Yeh-Li Ho, Beatriz Perondi, Jaques Sztajnbok, Venancio Avancini Ferreira Alves, Marisa Dolhnikoff, Edward C. Holmes, Paulo Hilário Nascimento Saldiva, Paolo Marinho de Andrade Zanotto

**Affiliations:** 1 Laboratory of Molecular Evolution and Bioinformatics, Department of Microbiology, Biomedical Sciences Institute, University of São Paulo, São Paulo, Brazil; 2 Pathology Department, Clinical Hospital, Faculty of Medicine, University of São Paulo, São Paulo, Brazil; 3 Faculty of Tropical AgriSciences, Czech University of Life Sciences in Prague, Prague, Czech Republic; 4 Intensive Care Unit, Division of Clinical Infectious and Parasitic Diseases, Clinical Hospital, Faculty of Medicine, University of São Paulo, São Paulo, Brazil; 5 Yellow Fever Crisis Committee, Clinical Hospital, Faculty of Medicine, University of São Paulo, São Paulo, Brazil; 6 Institute of Infectology Emílio Ribas, São Paulo, Brazil; 7 School of Life and Environmental Sciences & Sydney Medical School, The University of Sydney, Sydney, Australia; 8 Scientific Platform Pasteur - USP, São Paulo, Brazil; 9 Marie Bashir Institute for Infectious Diseases and Biosecurity, Charles Perkins Centre, School of Life & Environmental Sciences and Sydney Medical School, The University of Sydney, Sydney, NSW 2006 Australia

**Keywords:** Viral evolution, Viral transmission

## Abstract

The largest outbreak of yellow fever of the 21^st^ century in the Americas began in 2016, with intense circulation in the southeastern states of Brazil, particularly in sylvatic environments near densely populated areas including the metropolitan region of São Paulo city (MRSP) during 2017–2018. Herein, we describe the origin and molecular epidemiology of yellow fever virus (YFV) during this outbreak inferred from 36 full genome sequences taken from individuals who died following infection with zoonotic YFV. Our analysis revealed that these deaths were due to three genetic variants of sylvatic YFV that belong the South American I genotype and that were related to viruses previously isolated in 2017 from other locations in Brazil (Minas Gerais, Espírito Santo, Bahia and Rio de Janeiro states). Each variant represented an independent virus introduction into the MRSP. Phylogeographic and geopositioning analyses suggested that the virus moved around the peri-urban area without detectable human-to-human transmission, and towards the Atlantic rain forest causing human spill-over in nearby cities, yet in the absence of sustained viral transmission in the urban environment.

## Introduction

Yellow fever virus (YFV) is an enveloped virus of the family *Flaviviridae* (genus *Flavivirus*) with a single stranded, positive-sense RNA genome of approximately 11 kb that encodes a single polyprotein cleaved into three structural (capsid (C), membrane (M) and envelope (E)) and seven non-structural proteins (NS1-NS5)^1^. The virus comprises a single serotype with four genotypes: (*i*) East Africa, (*ii*) West Africa, (*iii*) South American I and (*iv*) South American II^2,3^ that may have diverged around several thousand years before present^4,5^ with a possible origin in the African continent^5,6^. Historical evidence points to a YFV introduction in the Americas around the 17^th^ century, possibly due to the slave trade^3,5–7^. After its introduction, YFV established both urban and sylvatic cycles^7,8^, and several urban outbreaks have been reported in Brazil since the 17^th^ century^9^. The circulation of YFV in the urban cycle in the American continent was initially mitigated by curbing the infestation of *Aedes aegypti* and later with the advent of an effective vaccine in the early 20^th^ century^7,10^, with considerable success. As a consequence, the last urban outbreak of YFV was officially reported in 1942 in Brazil^9^. After the reintroduction of *A. aegypti* in the 1970’s^11,12^ the virus remained, until recently, largely in sylvatic environments in the Americas, infecting non-human primates (NHPs) with sporadic cases in susceptible human hosts. The main vectors of YFV in the sylvatic cycle are mosquitoes of the genera *Haemagogus* and *Sabethes*^13,14^.

In 2014, intense enzootic activity of YFV was detected in Mato Grosso do Sul and Goiás states that adjoin the Amazon region of Brazil^15,16^. YFV carried by infected monkeys kept moving in a general southeasterly direction, and in 2016 cases were reported in Minas Gerais, reaching epidemic proportions in 2017, during which cases were also reported in the states of Rio de Janeiro, Espírito Santo and Bahia^17–21^.

Between January 2016 and January 2018, seven countries and regions of the Americas reported cases of yellow fever in their territories (Bolivia, Brazil, Colombia, Ecuador, French Guiana, Peru and Suriname), with the highest indices in Brazil. In early 2018 an unusually large increase in the number of confirmed cases was observed in the state of São Paulo^22^. A peak of notified human cases was reached in January 2018^23^. This was the largest outbreak registered in 21^st^ century in the most populated state of Brazil, including the densely populated metropolitan region of São Paulo city (MRSP), which is the largest conurbation in the southern hemisphere with around 23 million inhabitants. Until 2018, vaccination was not generally recommended in MRSP because YFV had been absent in recent decades. Hence, most of the population in the area was susceptible to YFV and autochthonous cases were reported^24,25^. Due to the outbreak in São Paulo, vaccination campaigns were initiated for resident populations, starting in northern peri-urban settings bordering forested and rural areas, where cases of YFV were previously reported. Subsequently, vaccination was extended to the whole urban population as well as to all inhabitants of the São Paulo state as the epidemic expanded^22^. As this is the first time in the 21^st^ century that cases of YFV have appeared in the MRSP, we sought to characterize the circulating viruses and establish their origin by studying their evolution and phylogeography based on samples taken from patients who died during the 2017–2018 outbreak.

## Material and Methods

### Ethical statement

The human autopsies analyzed in this study were performed after obtaining informed consent of the family members and following the protocol approved by the research ethics committee of the Clinical Hospital of the University of São Paulo School of Medicine (HCFMUSP) (CAPPesq #426.643). All the methods were performed in accordance with the relevant guidelines and regulations of the ethics committee of the HCFMUSP following the approval CAPPesq #426.643. All participating families were asked to sign a free and informed consent form, authorizing the autopsy and all experiments performed with the collected tissues. All laboratory procedures listed below were performed in a biosafety level (BSL)-2 laboratory, in accordance with the Brazilian standards of the Ministry of Health for Biological Agents Risk Classification^26^.

### Patients and samples

Overall, we analyzed 81 patients 67 of whom were confirmed to have died following YFV infection. We successfully acquired 36 genome sequences from the 67 yellow fever deaths, with the remaining samples being of insufficient quality to obtain YFV genomes at the necessary coverage. The suspected case definition of YFV infection was established by the Brazilian Ministry of Health and the Health Department of São Paulo State and included patients with sudden onset high fever associated with jaundice and/or hemorrhage who had lived or had visited areas with YFV epizootics (*i.e*., clusters of infections in non-human primates (NHP) or isolation of YFV in vectors), regardless of the vaccine status for YFV, during the preceding 15 days. Confirmed cases had compatible clinical presentation and laboratory confirmation by at least one of the following methods: (*i*) serum IgM positive (MAC-ELISA); (*ii*) detection of YFV-RNA by qRT-PCR in blood samples; (*iii*) virus isolation; (*iv*) histopathology compatible with YFV hepatitis with detectable antigen in tissues by immunohistochemistry technique. All cases received the definitive laboratorial diagnosis of YFV by the Adolfo Lutz Institute (IAL), the State Reference Laboratory. Previous exposure or co-infection by Hepatitis A virus (HVA), B (HBV), C (HVC), Cytomegalovirus (CMV), Herpes virus (HSV), Dengue virus (DENV), Chikungunya virus (CHIKV), Human Immunodeficiency virus type 1 (HIV-1), leptospirosis and other non-infectious diseases etiologies for acute hepatitis were accessed and cases were excluded following clinical diagnostic methods. Epidemiological, clinical (including demographic data, preexisting medical conditions, clinical signs and symptoms and in-hospital follow-up until death) and other laboratory features were collected from the medical charts.

### Autopsy protocol and tissue processing

The Service of Verification of Deaths of the Capital - USP investigated deaths due to yellow fever from December/2017 to April/2018. Autopsies were performed following the Letulle technique, where all the organs were removed *en masse* (one block), requiring dissection organ by organ to exam them individually. Briefly, the dissection was performed in the following organs: (*i*) heart; (*ii*) lung; (*iii*) brain; (*iv*) kidney; (*v*) spleen; (*vi*) pancreas; and (*vii*) liver.

### Molecular characterization

Nucleic acid extraction from all collected tissues was performed using the TRIzol® reagent (Life Technologies, Carlsbad, CA, USA) and carried out according to the manufacturer’s instructions. Molecular detection of YFV was performed with the use of the AgPath-ID One-Step RT-PCR Reagents (Ambion, Austin, TX, USA) with specific primers/probe previously described^27^. To identify cases of adverse vaccine response (*i.e*., fatal cases associated with the vaccine virus) we used specific primers/probe specific for the vaccine virus^28^. qRT-PCR reactions consisted of a step of reverse transcription at 45 °C for 10 min, enzyme activation at 95 °C for 10 min, and 40 cycles at 95 °C for 15 s and 60 °C for 45 s for hybridization and extension using the ABI7500 equipment (Thermo Fisher Scientific, Waltham, MA, USA).

### Sequencing and viral genome assembly

Based on the RNA viral concentration, total RNA were extracted from the liver tissues using the TRIzol® reagent (Life Technologies, Carlsbad, CA, USA). Subsequently, the RNA was purified with DNase I and concentrated using the RNA Clean and Concentrator ^TM-5^ kit (Zymo Research, Irvine, CA, USA) according to the manufacturer’s instructions. The paired-end RNA libraries were constructed and validated using the TruSeq Stranded Total RNA HT sample prep kit (Illumina, San Diego, CA, USA). Sequencing was done at the Core Facility for Scientific Research – University of São Paulo (CEFAP-USP/GENIAL) using the Illumina NextSeq platform. Each sample was barcoded individually, which allowed separation of reads for each patient. Short unpaired reads and low-quality bases and reads were removed using Trimmomatic version 0.36 (LEADING:20 TRAILING:20 SLIDINGWINDOW:4:25 MINLEN:36)^29^. Consensus genomes were assembled with paired-end reads using Bowtie2 v.2.3.4.3^30^ using default parameters.

### Data sets

All full genomic sequences available from YFV that contained information on location and date of isolation were recovered from the National Center for Biotechnology Information (NCBI) (https://www.ncbi.nlm.nih.gov/genbank/) website. Sequences were aligned to our 36 new YFV genomes (Supplementary Table 1) using Clustal Omega v.1.2.4^31^. A list of the sequences used is available in Supplementary Table 2. Recombinant sequences were screened using all algorithms implemented in RDP4 program (RDP, GENECONV, BootScan, MaxChi, Chimaera, Siscan and 3Seq) using the default settings^32^. No evidence for recombination was detected. Sequences containing long contiguous stretches of undefined nucleotides were excluded. A final alignment of complete genome sequences was manually inspected and edited using the program AliView v.1.18^33^. After preliminary phylogenetic analyses, the master alignment comprising 135 full-length, curated sequences encoding the complete viral polyprotein (dataset-1) (Supplementary Table 2) was subdivided into two data sets for further analysis: (*i*) a data set containing 98 genomes of the SA1 and SA2 genotypes from the Americas (dataset-2); and (*ii*) 74 sequences from 2017 and 2018 sampled from the states of Minas Gerais, Espírito Santo, Bahia, Rio de Janeiro and São Paulo (dataset-3) (Supplementary Tables 2 and 3). All alignments are available in the Supplementary Data and on GitHub (https://github.com/MarieltonCunha/ViralDiversity/).

### Phylogenetic analysis

Phylogenetic trees of YFV based on full-length, curated coding sequences for all the data sets were estimated using the Maximum Likelihood (ML) method implemented in IQ-TREE 1.5.5^34^ with automatic model selection by ModelFinder and using the Bayesian Information Criterion (BIC)^35^. The robustness of the groupings observed was assessed using 1,000 non-parametric bootstrap replicates. ML and Bayesian maximum clade credibility (MCC) trees (see below) were visualized and plotted using FigTree v.1.4.3^36^. All taxon labels for sequences used in this work are presented in the format: genotype/accession number/strain name/local of isolation/date of isolation. We explored the temporal signal (*i.e*., molecular clock structure) and quality of our data set using TempEst v.1.5.1^37^.

### Phylodynamics and phylogeographic analysis

The spatio-temporal evolution of YFV spread was inferred within a Bayesian framework as implemented in BEAST v.1.10.1^38^. An initial descriptive summary of the demographic history of YFV was approximated using the Bayesian SkyGrid coalescent model^39^ and revealed no significant variation in genetic diversity (a marker of population size) during the period of our analysis. Based on previous estimates of evolutionary dynamics of related YFV^17,40^, we tested uncorrelated relaxed molecular clocks assuming a log-normal distribution, in combination with constant size, exponential and logistic growth demographic models (Supplementary Tables 4 and 5). Phylogeographic patterns and parameters were estimated using the Bayesian inference through Markov chain Monte Carlo (MCMC) run for 50 million states, sampling every 5,000 states with a 10% burn-in. Convergence and the effective sample size (ESS) > 200 were examined using Tracer v.1.7.1^41^. Likewise, the maximum clade credibility tree (MCC) was visualized and edited in FigTree v.1.4.3^36^. We recorded the time to the most recent common ancestor (tMRCA) and their 95% Bayesian credible intervals (HPD) for the MCC tree. To calculate the log marginal likelihood for molecular clock and demographic model selection, we used the path sampling (PS) and the stepping-stone (SS) sampling approaches by running 100 path steps of 1 million iterations each^42^. The spatiotemporal spread of YFV was visualized and plotted with SPREAD3^43^. XML input files for BEAST are available in the Supplementary Data and on GitHub (https://github.com/MarieltonCunha/ViralDiversity/).

### Geopositioning of samples

To analyze the geographical proximity among fatal human and NHP cases we calculated the spatial distances between all cases using available geoposition information. We geopositioned only those fatal human and NHP YFV cases that occurred in the MRSP (47.0–46.2 S, 23.9–23.1 W), using the available data on patient residence and day of death. NHP cases were included only for those were coordinates for the place of where carcasses were found was available. For fatal NHP cases, the date the carcass was found was assumed to be the day of death, although death may have taken place a few days before. Distances between the human and NHP fatal YFV cases were calculated based on the available coordinates. Geographic pairwise distance matrices among all YFV cases (in kilometers) were clustered using the neighbor joining algorithm available in the PHYLIP v.3.695 package^44^, this enabled us to produce a dendogram based on geoposition information.

## Results

### Epidemiological surveillance of YFV in São Paulo, 2017–2018

**Figure 1:**
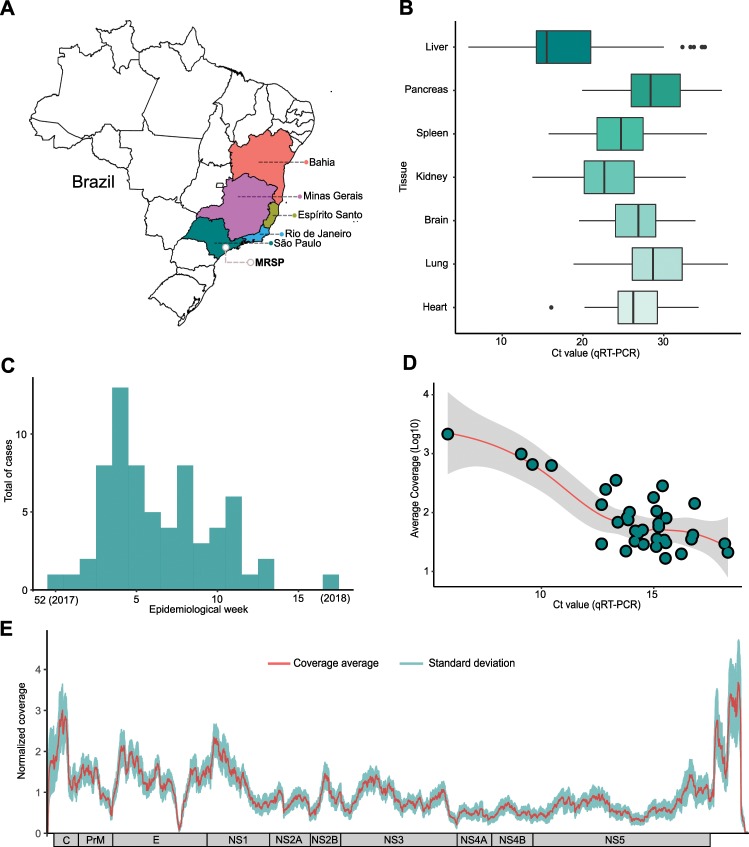
The current outbreak of yellow fever virus in Brazil (2016–2018). (**A**) Brazilian states with YFV cases recorded and sequenced in humans, non-human primates (NHP) and mosquitoes between 2017–2018. A grey circle marks the metropolitan region of São Paulo (MRSP). (**B**) Cycle threshold according to each of the 7 tissues analyzed for positive patients. Boxplots represent the 75th percentile, median, 25th percentile and the whiskers extend to the highest and lowest value in the 1.5x interquartile range. The different colors represent the different tissues analyzed. (**C**) Total cases recorded represented sylvatic cases of YFV (qRT-PCR positive cases) during the epidemiological weeks covered by the study (week 52 of 2017 to week 17 of 2018). (**D**) Relationship between the average coverage and the Ct values obtained for each sequenced sample. The data indicate that we obtained the expected direct inverse relationship between Ct and coverage parameters, as indicated by the trend line. (**E**) Combined coverage (normalized by the sample average) along all 36 sequenced YFV genomes generated in this study.

From January to August 17, 2018, the State of São Paulo reported 3028 suspected cases of yellow fever, 537 (17.7%) of which were confirmed, with 498 (92.7%) autochthonous cases and 35 (6.5%) imported from other states^45^. Of the 498 autochthonous cases, 176 died, resulting in a mortality frequency of 35.4%^45^. Despite the magnitude of the outbreak in São Paulo, little is known about the epidemiological, genetic and evolutionary characteristics of the virus circulating in the state. Accordingly, among all patients who died with suspected YFV infection between December 2017 and April 2018, we focused on 81 cases identified through the service of verification of deaths of the capital - USP (SVOC-USP) in the city of São Paulo (Fig. 1A). Our qRT-PCR results indicated that 67/81 (82.7%) individuals had been infected by YFV, while five were shown by qRT-PCR to only carry the vaccine strain YFV-17DD alone, suggesting that their death was associated with an adverse response to the vaccine as previously reported^46–48^, and nine were negative for YFV infection in all tissues tested (Fig. 1B,C). All 67 confirmed YFV deaths were due to complications of fulminant yellow fever hepatitis, with hepatic encephalopathy, severe coagulopathy, bleeding (mainly gastrointestinal, pulmonary and/or cerebral hemorrhages), renal dysfunction and secondary infections. We were able to successfully sequence the full YFV genome from 36 of these patient samples.

All of our cases were sampled in 17 localities in the São Paulo state, from which 16 localities had fatal cases due to YFV (Supplementary Table 6). Our molecular diagnostics indicated a peak of cases during the first epidemiological weeks of 2018, particularly at the end of January, coinciding with official cases notifications data (Fig. 1C). The median age of people with confirmed infection was 49.12 years (range 16–87) and were mainly male (82.09–55/67).

### Genomic surveillance

**Figure 2:**
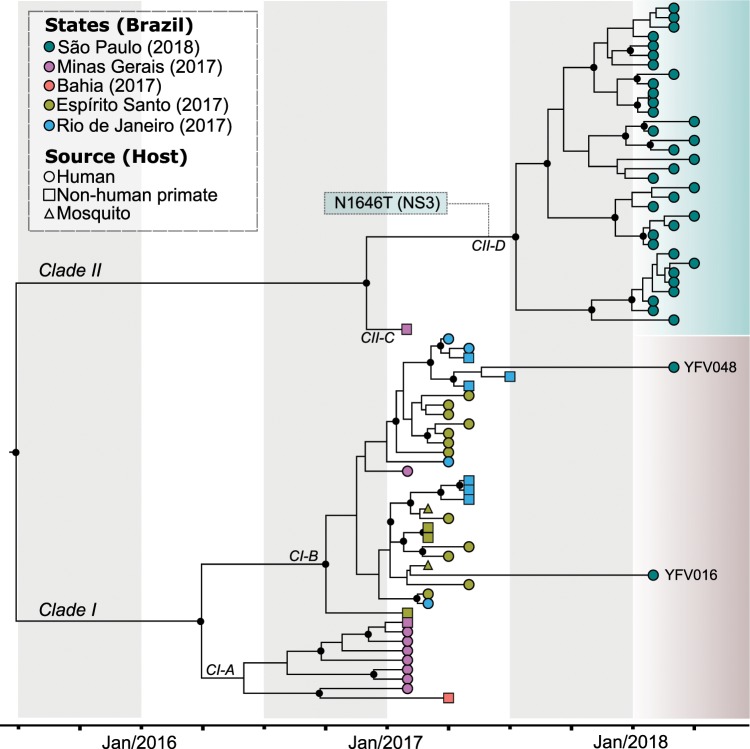
Time-stamped, MCC tree of YFV South American genotype I in Brazil recovered under the logistic-lognormal demographic model. The different colours indicate samples from different locations. The black circles represent posterior support upper than 0.7. The single synapomorphic change observed in Clade II [N1646T (NS3)] is shown in the box over the branch leading to Clade II-D. The three distinct introductions in the metropolitan region of São Paulo (MRSP) are shown (See also Fig. 3).

**Figure 3:**
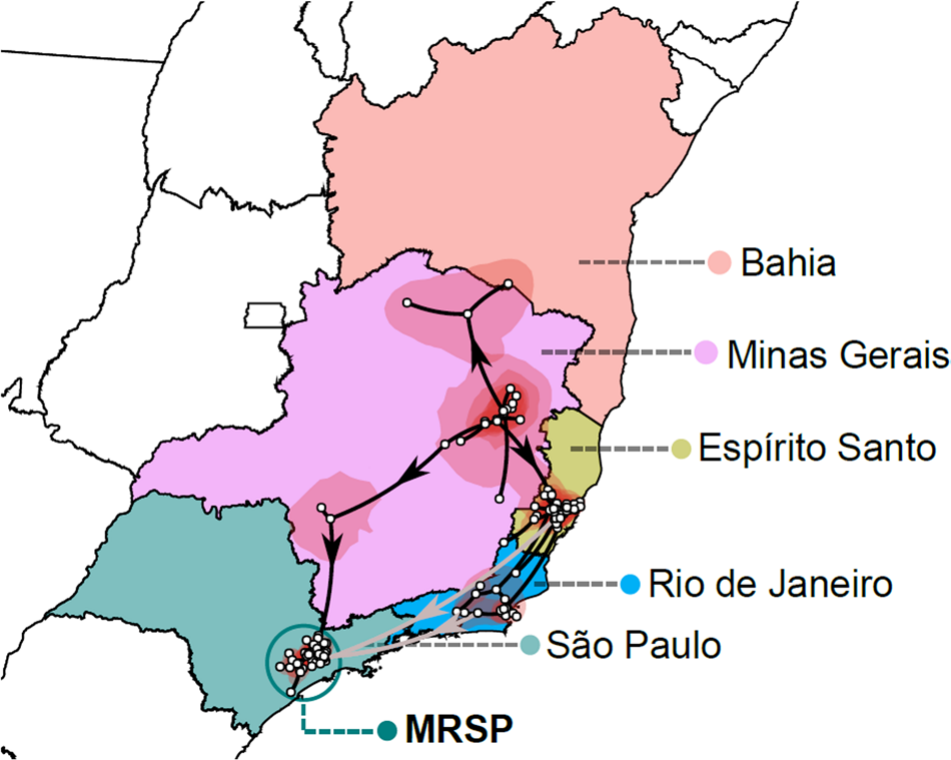
Highest posterior probability migration paths for the YFV Clades I and II from 2016 to 2018 towards the metropolitan region of São Paulo (MRSP), based on the analysis of 74 complete genomes. Although the sample size is small such that inferences should be made with caution, three distinct introductions in the MRSP are shown and strongly supported. The spatiotemporal spread was visualized with SPREAD3.

Because detailed spatio-temporal resolution of viral evolution often relies on a few nucleotide differences among otherwise closely related viruses, complete genomes with high coverage for each base position are a prerequisite for robust inference. Therefore, to select the appropriate clinical specimens for viral sequencing, we analyzed cycle threshold (Ct) data from qRT-PCR from viral RNA in seven distinct tissues/organs (heart, lung, brain, kidney, spleen, pancreas and liver) to choose samples with the lowest possible Cts. In general, all tissues had normally distributed Ct values, with the exception of the liver, which had a moderately asymmetrical distribution and a deviation to lower Ct values, and hence generally inferior to other tissues (Fig. 1B). In total, we obtained 36 complete YFV genomes from the 67 positive patients (Fig. 1D,E). All sequences of the current outbreak belonged to the South American I genotype (Supplementary Fig. 1), and were related with sequences previously isolated in neighboring states in 2017 (Fig. 2) with no evidence of recombination. Based on the phylogenetic analysis, we could infer at least three distinct introductions of YFV in the MRSP: (*i*) A major clade (34 genomes) in the northwest of the MRSP coming from Minas Gerais due to NHP movement, and likely emerging between April 2017–October 2017 (95% HPD; mean - July 2017) (Fig. 3 and Supplementary Tables 4 and 5), (*ii*) one virus lineage from a case from Espírito Santo (Patient 16), and (*iii*) one from a case from Rio de Janeiro (Patient 48) (Fig. 2). Importantly, our patient’s records indicated the two single introductions were due to people visiting enzootic locations in these states and did not appear to have caused detectable additional cases in the MRSP.

### Origin of the 2016–ongoing Yellow Fever virus outbreak

Phylogenetic (Supplementary Figs. 1 and 2) and phylogeographic (Figs. 2, 3 and Supplementary Tables 4 and 5) analyses of samples from the 2017–2018 YFV outbreak allowed us to reveal the origin and spread of YFV in the Southeast and Northeast region of Brazil. In particular, there was evidence of two distinct zoonotic clades (Clade I and II) that likely separated in Minas Gerais (location posterior support of 0.8) between November 2013–June 2016 (95% HPD; mean date of June 2015). The mean rate of Clade I and II migration during the whole sampled period 2017 to 2018 was approximately 3.3 km/day (95% HPD = 2.25–4.37 km/day) with a mean evolutionary rate of 9.85 × 10^−4^ nucleotide substitutions per site, per year (subs/site/year) (95% HPD = 6.52 × 10^−4^ − 1.35 × 10^−3^ subs/site/year). We now describe these two clades in more detail.

### Clade I

Clade I divided into two smaller clades (CI-A and CI-B) in 2016 (95% HPD of divergence time = July 2015 – September 2016) and likely in Minas Gerais (location posterior support of 0.82) (Fig. 2). CI-A then diversified and moved and into peri-urban and forested regions in the state of Minas Gerais, causing an outbreak after January 2017, then moving onto Bahia. In contrast, Clade CI-B likely diversified in the forest region in the border between Minas Gerais and Espírito Santo, also in 2016, and then moved to Espírito Santo and Rio de Janeiro, causing in both states an outbreak during the first part of 2017. Two YFV patients who died in 2018 and resided in the MRSP had visited Espírito Santo (Patient 16) and Rio de Janeiro (patient 48). Fittingly, the virus phylogeny showed that their posthumous viral samples were nested among isolated viruses from the areas they visited (Fig. 2). These results indicated that CI-B was circulating until early 2018.

### Clade II

**Figure 4:**
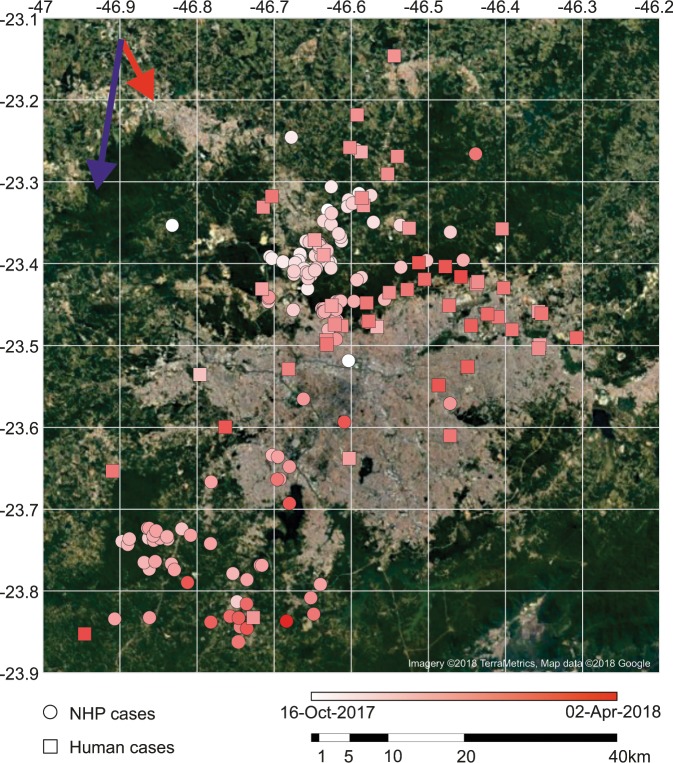
Spatial distribution of YFV deaths through time in non-human primates (NHP) and humans. Arrows indicate the general trend of movement around the metropolitan region of São Paulo (MRSP) estimated from distance matrices (see Fig. 5). The earliest cases in NHP are shown in the north, and later in the south and northeast of the MRSP. Most human cases are near sites with reported deaths of NPH, confirmed to be caused by YFV. The outbreak appears to have been confined mostly near the forested belt around the MRSP, contrasting with the almost empty, heavily urbanized center. Cardinal points are aligned according to the main axis of the page, (*e.g*., top being north, etc.). The figure was created by plotting the coordinates of reported cases to a satellite image available from Google Maps (google.com/maps) as background.

This clade caused the majority of the deaths in the MRSP (Fig. 2). It diverged into Clades CII-C and CII-D in the state of Minas Gerais, with a location posterior support of 0.87, near the border with São Paulo between June 2016 - January 2017 (95% HPD; mean - December 2016) (Fig. 3). Subsequently, CII-D moved towards the MRSP, causing epizootics beginning between April 2017–October 2017 (95% HPD; mean - July 2017) (Supplementary Fig. 3) in forest parks (Horto Florestal and Cantareira State Park) that form a belt around the Northern part of the MRSP (Fig. 4). It is noteworthy that our inferred dates correspond well with the reported official cases of YFV cases in NHP and humans (Fig. 4). It is also notable that CII-D is also defined by a unique synapomorphic substitution (N1646T) in the NS3 gene that is not present in CII-C and Clade I viruses (Fig. 2).

### Geopositioning analysis

**Figure 5:**
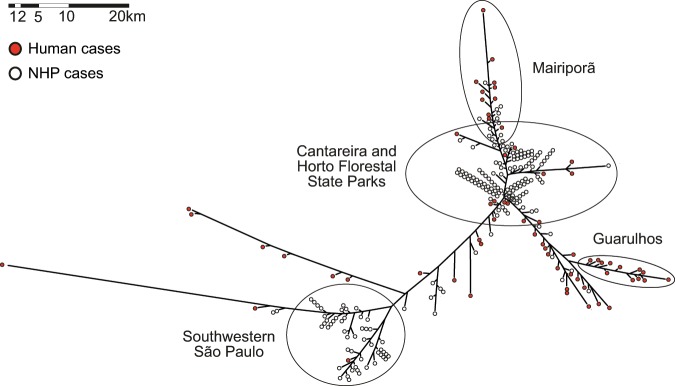
Neighbor joining tree calculated from pairwise geoposition distances among all the non-human primates and human cases available from the metropolitan region of São Paulo (MRSP).

In total, 230 NHP carcasses were collected in the MRSP. Of these, 136 were members of the genus *Alouatta* (howler monkeys), 14 were *Callithrix* genus (marmosets), and five were *Cebus* genus (capuchin monkeys). The species identity of the remaining 75 carcasses were not determined (Fig. 4) (data provided by the Adolfo Lutz Institute). Analysis of spatio-temporal data showed that the YFV outbreak progressed in different directions in humans and NHPs (Figs. 4 and 5). While the outbreak in NHPs had a tendency to move in a south-southwest direction, in humans the outbreaks in a southeast direction (Figs. 4 and 5).

Several geographically well-defined clusters can be observed in the dendogram inferred from the pairwise geographic distances matrix among all YFV cases (Fig. 5). Two areas of intense epizootics were inferred in the north and southwest forested areas around the MRSP. We also inferred a large cluster of cases of NHP and humans in the northern region, Cantareira and Horto Florestal State parks, spreading to the nearby towns of Mairiporã and Guarulhos, where most of the human and NHP cases were reported. Another cluster represents NHP cases from the southwestern of the MRSP, around Cotia, where the second most affected NHP population was present. Hence, the most striking finding of this analysis was that most human cases occurred close to both the NPH cases and the forested belt around the MRSP.

## Discussion

We describe the outbreak of YFV in the MRSP, Brazil, in 2016–2018, particularly its origin and how the virus diversified and moved around the largest conurbation in the southern hemisphere carried by NHP, killing 176 people during 2018 in the process^45^. All the isolates from São Paulo belonged to the South American I genotype and formed a single monophyletic group along with viruses (comprising Clades I and II) that also circulated in 2016–2017 in the states of Minas Gerais, Bahia, Rio de Janeiro and Espírito Santo^17,19,40^. Several synapomorphic mutational changes in different genes were previously reported by our group^19^, and here we report a synapomorphy (N1646T) in the protease NS3 gene shared by all CII-D. The mean evolutionary rate for all the YFV sequences of the Brazilian outbreak (2017–2018) was 9.85 × 10^−4^ subs/site/year, and hence compatible with those previously estimated for YFV and for other flaviviruses^4,5,49^.

The current Brazilian outbreak began in the state of Minas Gerais in June 2015, with all viruses sampled from 2017 belonging to a single monophyletic group that diverged into two main clades (Clade I and II), and indicative of a single introduction of the virus in the region. These observations are supported by other molecular epidemiological studies conducted in Brazil from 2016^17–19,40,50^. Although previous evolutionary studies point to an origin of the virus in Venezuela^40^, epidemiological monitoring carried out by the Brazilian Ministry of Health suggest a likely origin of the Brazilian outbreak in 2014, with confirmed epizootics in the transitional area between the Amazon and the Cerrado biomes (with most of the confirmed cases occurring in the states of Goiás and Mato Grosso do Sul)^15,16,21^. This region was the probable link between the Amazon basin and the state of Minas Gerais, located in southeastern Brazil. It is likely that the numbers of human cases in this region were not high due to the vaccine coverage there^51^. The viral invasion into southeast Brazil, associated with the rapid spatial spread of the virus (estimated here at a mean of rate 3.3 km/day), caused the virus to circulate in important fragments of the Atlantic Forest near the peri-urban areas of the main Brazilian megacities (notably São Paulo and Rio de Janeiro), and led to a marked increase in the number of cases during the outbreak. In the MRSP, the virus (Clade CII-D) was introduced, maintained and spread in the sylvatic transmission cycle, with occasional cases of infection in humans between April 2017 and October 2017, with the interstate border between São Paulo and Minas Gerais as the route of introduction. In São Paulo state, the routes of viral dispersion included only interconnected forested, corridors linked to peri-urban regions. The patients studied here were mainly unvaccinated adult males that had contact with the sylvatic environment or lived nearby. No autochthonous cases were documented in the central region of the city of São Paulo. Importantly, the MRSP cases reduced in numbers as the populations of NHP collapsed and with vaccination campaigns in areas classified as at risk^52^.

The introduction and establishment of the YFV Clade II-D in the state of São Paulo can be further explained by environmental factors, including: (*i*) mosquitoes of the genus *Haemagogus* are abundant in the forested areas of the state of São Paulo^53,54^ and were the primary vectors in the YFV outbreak occurred in Brazil, 2016–2018^55^; (*ii*) NHPs are found in areas of the Atlantic Forest and are susceptible and responsible for the maintenance of the virus in the sylvatic cycle^56–58^; and (*iii*) the regions affected by the current outbreak had low vaccine coverage^51^. Our findings support previous work indicating that the outbreak of 2016–2018 (sampled in the states of Minas Gerais, Bahia, Espírito Santo and Rio de Janeiro), occurred in a sylvatic environment with occasional infections in humans^17^.

Importantly, we also recorded two introductions of YFV Clade I-B detected in patients who travelled to Espírito Santo and Rio de Janeiro - both states that experienced significant circulation of this virus lineage in 2018. In both these states an increase in the number of YFV notifications was reported in 2017 across successive epidemic periods, showcasing the maintenance of epizootic YFV. In addition, we highlighted the extent of viral movement, such as observed in cases imported from Brazil by other countries^59^, largely facilitated by rapid human movement such as those resulting from air travel^60^.

In contrast to other arboviruses in Brazil such as dengue virus, in which continuous reintroductions are responsible for keeping the virus circulating in the urban cycle^61–63^, YFV is dependent on epizootics to cause cases in humans. The South American I genotype belongs to a “modern lineage”, that has been circulating in America since 1995 and that perhaps originated in Trinidad and Tobago^40^. It is believed that from there the virus spread to South American countries, especially Venezuela and Brazil^40^, carried mainly by NHP and sylvatic mosquitoes, moving along forested corridors and perhaps promoted by a series of interlocked epizootics involving the exchange of viruses among infected and susceptible individuals^64,65^. Epizootics among social animals, such as New World arboreal primates, may be reduced by self-exclusion of infected individuals^66^. For instance, it is in theory possible that social avoidance, changes in group size, group isolation and several other behaviors may have evolved due to reduce pathogen transmission. Nevertheless, in the case of vector-borne diseases any isolation mechanism is efficient only at distances that minimize transmission^66^. Howlers were the most affected monkey species in the forested belt around the MRSP^52^. As in several other previous YFV epizootics^64^, the high overall fatality rate in howlers led to almost the complete extinction of these monkeys in most areas around Sao Paulo^52^.

It has been assumed that the decline in the numbers of howler monkeys and the severe reduction of several species of NHP from around the MRSP had a significant effect on ending the outbreak. Although perhaps due to poor sampling of monkeys in that locality, it is possible that Clade II-D could have caused a limited number of human-to-human transmission cases, as suggested by a cluster of human cases in Guarulhos (Fig. 5). Critically, however, a key factor that differentiates the current outbreaks of YFV in the Americas and Africa is that there is no clear evidence for urban cycles of YFV in the Americas has been observed since the first half of the 20^th^ Century. A possible, although untested, explanation is that the former *A. aegypti* colonizing the Americas was from Africa (Senegalese strain), while the *A. aegypti* reintroduced in the early 1970’s is Asiatic, where no urban spread of YFV is observed^67^.

## Supplementary information

Supplementary information.

Supplementary dataset.
