# Supplementary material for: Origin of the São Paulo Yellow Fever epidemic of 2017–2018 revealed through molecular epidemiological analysis of fatal cases
Source: Sci Rep. 2019 Dec 31;9:20418. doi: 10.1038/s41598-019-56650-1 (PMC6938505; doi:10.1038/s41598-019-56650-1)
Supplement: Supplementary file 1 — Supplementary information. [file 41598_2019_56650_MOESM1_ESM.pdf]

# Supplementary information for:

# Origin of the São Paulo Yellow Fever epidemic of 2017-2018 revealed through molecular epidemiological analysis of fatal cases

Marielton dos Passos Cunha*, Amaro Nunes Duarte-Neto, Shahab Zaki Pour, Ayda Susana Ortiz-Baez, Jiří Černý, Bárbara Brito de Souza Pereira, Carla Torres Braconi, Yeh-Li Ho, Beatriz Perondi, Jaques Sztajnbok, Venancio Avancini Ferreira Alves, Marisa Dolhnikoff, Edward C. Holmes, Paulo Hilário Nascimento Saldiva, Paolo Marinho de Andrade Zanotto*

* **Corresponding authors**: marieltondospassos@gmail.com (M.P.C.); pzanotto@usp.br (P.M.A.Z.)

**Contents:**

- Supplementary Figure 1
- Supplementary Figure 2
- Supplementary Figure 3
- Supplementary Table 1
- Supplementary Table 2
- Supplementary Table 3
- Supplementary Table 4
- Supplementary Table 5
- Supplementary Table 6

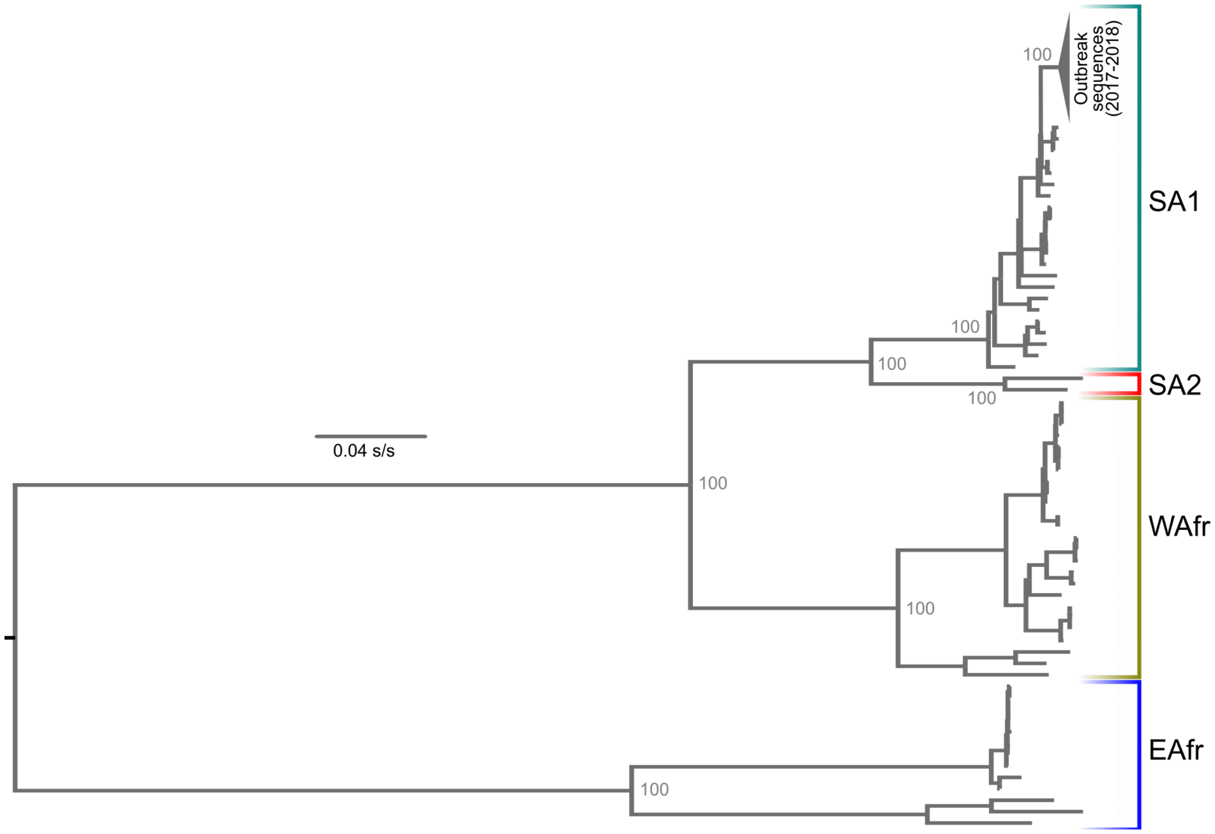


**Supplementary Figure 1.** Maximum likelihood phylogenetic trees for YFV based on full-length genome sequences (n = 135). The tree is midpoint-rooted and nodes are labelled with bootstrap support values. The distinct colors represent distinct genotypes: (*i*) EAfr - East African; (*ii*) WAfr - West African; (*iii*) SA1 - South American I; (*iv*) SA2 - South American II.

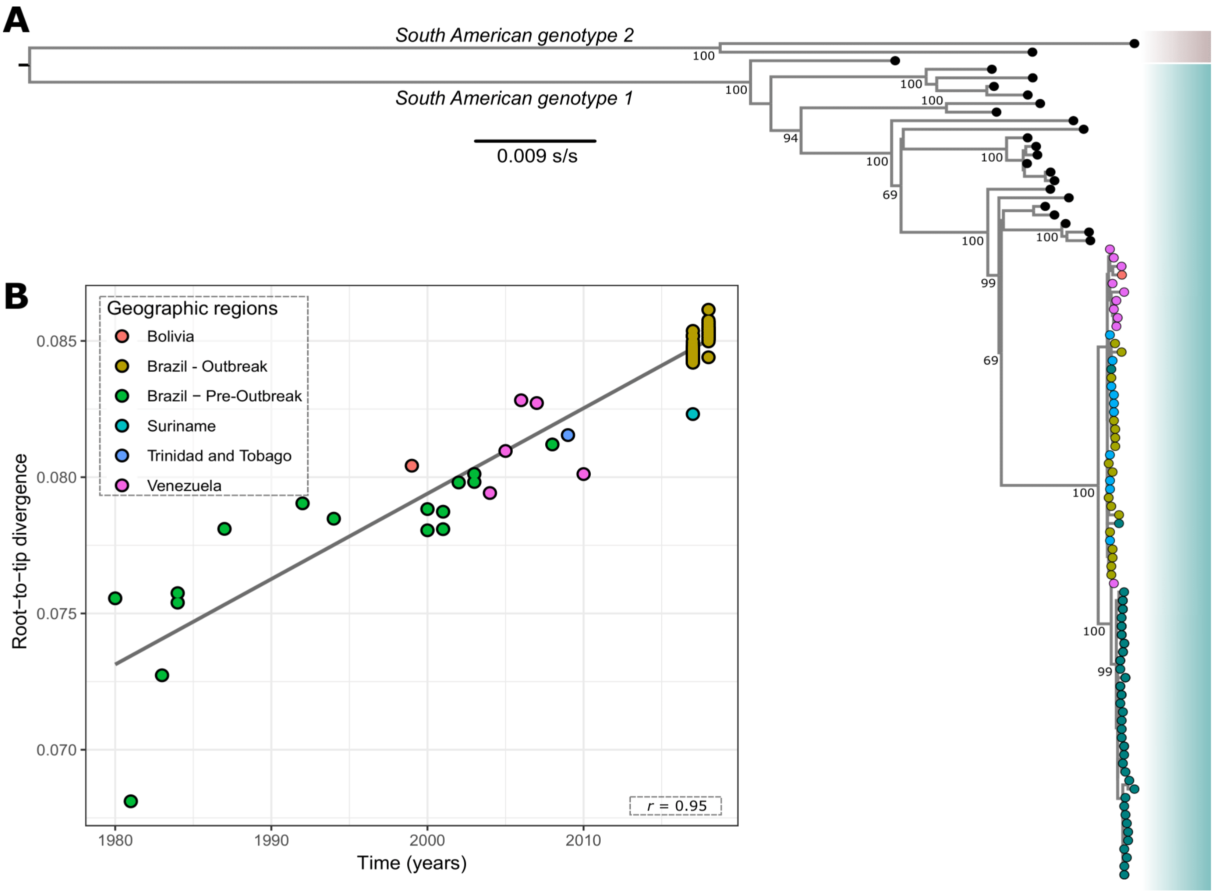


**Supplementary Figure 2.** (A) Maximum Likelihood phylogenetic trees for South American (genotype I and II) YFV based on full-length genome sequences (n = 98). The tree is midpoint-rooted and nodes are labelled with bootstrap support values. The distinct colors represent distinct genotypes: (*i*) South American I and (*ii*) South American II. (B) A regression of root-to-tip genetic distance against time of sampling and showing a positive relationship (r = 0.95) indicative of a high rate of evolutionary change over the sampling period.

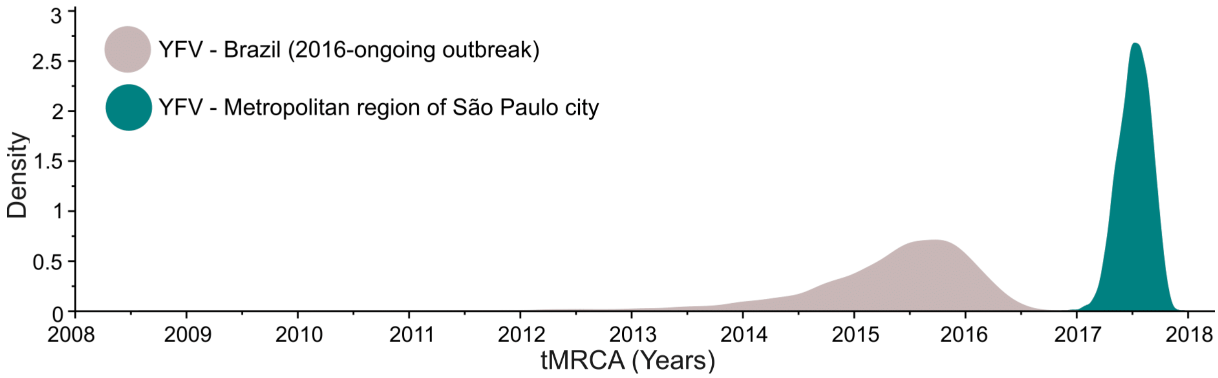


**Supplementary Figure 3**. Highest posterior probability density distribution for the time to the most recent common ancestor (tMRCA) estimated for the outbreak (green) and for the metropolitan region of São Paulo (MRSP) (gray).

**Supplementary Table 1.** Complete genomes sequences of human samples from the metropolitan region of São Paulo (MRSP).

| Strain name | GenBank number | Tissue | Local of isolation | Date of isolation | Ct | Coverage |
|---|---|---|---|---|---|---|
| YFV003FIG | MK583147 | Liver | Mairiporã | 12 January 2018 | 16.23 | 19.8633 |
| YFV004FIG | MK583148 | Liver | Mairiporã | 13 January 2018 | 15.39 | 283.649 |
| YFV005FIG | MK583149 | Liver | São Paulo | 16 January 2018 | 10.45 | 627.765 |
| YFV007FIG | MK583150 | Liver | Itaquaquecetuba | 15 January 2018 | 13.86 | 74.9691 |
| YFV008FIG | MK583151 | Liver | Mairiporã | 15 January 2018 | 15.55 | 80.1772 |
| YFV010FIG | MK583152 | Liver | Ibiúna | 16 January 2017 | 12.68 | 29.1631 |
| YFV015FIG | MK583153 | Liver | Mairiporã | 20 January 2018 | 12.68 | 136.145 |
| YFV016FIG | MK583154 | Liver | São Paulo | 21 January 2018 | 13.92 | 101.29 |
| YFV018FIG | MK583155 | Liver | Mairiporã | 22 January 2018 | 15.47 | 34.1318 |
| YFV020FIG | MK583156 | Liver | Atibaia | 23 January 2018 | 15.11 | 26.4692 |
| YFV021FIG | MK583157 | Liver | Guarulhos | 23 January 2018 | 15.52 | 16.6804 |
| YFV022FIG | MK583158 | Liver | Mairiporã | 23 January 2018 | 14.18 | 48.7968 |
| YFV023FIG | MK583159 | Liver | Atibaia | 24 January 2018 | 9.1 | 984.74 |
| YFV029FIG | MK583160 | Liver | Cotia | 29 January 2018 | 13.76 | 22.1379 |
| YFV033FIG | MK583161 | Liver | Itaquaquecetuba | 24 January 2018 | 14.16 | 32.5673 |
| YFV038FIG | MK583162 | Liver | São Paulo | 31 January 2018 | 9.6 | 653.547 |
| YFV040FIG | MK583163 | Liver | São Paulo | 02 February 2018 | 15.14 | 35.9072 |
| YFV041FIG | MK583164 | Liver | Cotia | 03 February 2018 | 12.86 | 246.849 |
| YFV042FIG | MK583165 | Liver | Mairiporã | 04 February 2018 | 16.82 | 142.497 |
| YFV043FIG | MK583166 | Liver | São Paulo | 06 February 2018 | 15.22 | 65.3013 |
| YFV047FIG | MK583167 | Liver | Guarulhos | 11 February 2018 | 14.3 | 44.3845 |
| YFV048FIG | MK583168 | Liver | São Paulo | 11 February 2018 | 16.74 | 41.6075 |
| YFV052FIG | MK583169 | Liver | Guarulhos | 18 February 2018 | 18.16 | 29.569 |
| YFV053FIG | MK583170 | Liver | Guarulhos | 18 February 2018 | 5.83 | 2143.13 |
| YFV054FIG | MK583171 | Liver | Guarulhos | 18 February 2018 | 16.68 | 35.2501 |
| YFV056FIG | MK583172 | Liver | Guarulhos | 19 February 2018 | 14.99 | 180.059 |
| YFV060FIG | MK583173 | Liver | Guarulhos | 23 February 2018 | 14.5 | 50.6066 |
| YFV061FIG | MK583174 | Liver | Arujá | 24 February 2018 | 15.54 | 30.6265 |
| YFV065FIG | MK583175 | Liver | Itaquaquecetuba | 27 February 2018 | 12.68 | 29.4161 |
| YFV066FIG | MK583176 | Liver | Piedade | 27 February 2018 | 14.54 | 28.8237 |
| YFV070FIG | MK583177 | Liver | Guarulhos | 04 March 2018 | 13.82 | 82.4984 |
| YFV072FIG | MK583178 | Liver | Itariri | 10 March 2018 | 13.4 | 68.4612 |
| YFV074FIG | MK583179 | Liver | Ibiúna | 11 March 2018 | 13.32 | 352.811 |
| YFV076FIG | MK583180 | Liver | Guarulhos | 14 March 2018 | 15.13 | 105.489 |
| YFV080FIG | MK583181 | Liver | São Lourenço da Serra | 27 March 2018 | 15.22 | 57.1338 |
| YFV081FIG | MK583182 | Liver | Guarulhos | 14 March 2018 | 18.3 | 21.0936 |

**Supplementary Table 2.** Complete YFV genomes sequences used in the phylogenetic analysis.

| Strain name | GenBank number | Location | Year | Genotype |
|---|---|---|---|---|
| BeAn754036 | KY861728 | Brazil - Rio Grande do Sul | 2008 | South American I |
| 8582H | U54798 | Côte d'Ivoire | 1982 | West African |
| 17DDBrazil | DQ100292 | Brazil | 1975 | West African |
| Uganda48a | AY968065 | Uganda | 1948 | East African |
| Couma | DQ235229 | Ethiopia | 1961 | East African |
| Uganda2010 | JN620362 | Uganda | 2010 | East African |
| Angola71 | AY968064 | Angola | 1971 | East African |
| CIC1 | KX010994 | China | 2016 | East African |
| Ogbomosho | KU978763 | Nigeria | 1946 | West African |
| YFV112 | MF465805 | Brazil - Minas Gerais | 2017 | South American I |
| ES505 | KY885001 | Brazil - Espírito Santo | 2017 | South American I |
| M211 | MH018096 | Brazil - Minas Gerais | 2017 | South American I |
| M210 | MH018095 | Brazil - Minas Gerais | 2017 | South American I |
| M138 | MH018093 | Brazil - Minas Gerais | 2017 | South American I |
| M123 | MH018092 | Brazil - Minas Gerais | 2017 | South American I |
| M68 | MH018080 | Brazil - Minas Gerais | 2017 | South American I |
| M58 | MH018079 | Brazil - Minas Gerais | 2017 | South American I |
| M51 | MH018078 | Brazil - Minas Gerais | 2017 | South American I |
| 3495 | MF170981 | Brazil - Espírito Santo | 2017 | South American I |
| 2563 | MF170980 | Brazil - Espírito Santo | 2017 | South American I |
| 3472 | MF170979 | Brazil - Espírito Santo | 2017 | South American I |
| 2841 | MF170978 | Brazil - Espírito Santo | 2017 | South American I |
| 1122 | MF170977 | Brazil - Espírito Santo | 2017 | South American I |
| 282 | MF170976 | Brazil - Espírito Santo | 2017 | South American I |
| 1555 | MF170975 | Brazil - Espírito Santo | 2017 | South American I |
| 3919 | MF170973 | Brazil - Espírito Santo | 2017 | South American I |
| 2992 | MF170972 | Brazil - Espírito Santo | 2017 | South American I |
| m6590PNH | MF170971 | Brazil - Minas Gerais | 2017 | South American I |
| es2487 | MF170970 | Brazil - Espírito Santo | 2017 | South American I |
| rj1930 | MF170969 | Brazil - Espírito Santo | 2017 | South American I |
| 3925 | MF170968 | Brazil - Espírito Santo | 2017 | South American I |
| RJ104 | MF538786 | Brazil - Rio de Janeiro | 2017 | South American I |
| RJ97 | MF538785 | Brazil - Rio de Janeiro | 2017 | South American I |
| H196 | MF538784 | Brazil - Rio de Janeiro | 2017 | South American I |
| H191 | MF538783 | Brazil - Rio de Janeiro | 2017 | South American I |
| H190 | MF538782 | Brazil - Rio de Janeiro | 2017 | South American I |
| RJ96 | MF423378 | Brazil - Rio de Janeiro | 2017 | South American I |
| RJ95 | MF423377 | Brazil - Rio de Janeiro | 2017 | South American I |
| RJ94 | MF423376 | Brazil - Rio de Janeiro | 2017 | South American I |
| RJ87 | MF423375 | Brazil - Rio de Janeiro | 2017 | South American I |
| PA196 | MF423374 | Brazil - Espírito Santo | 2017 | South American I |
| PA193 | MF423373 | Brazil - Espírito Santo | 2017 | South American I |

| | | | | |
|---|---|---|---|---|
| H199 | MF434851 | Brazil - Rio de Janeiro | 2017 | South American I |
| ES504 | KY885000 | Brazil - Espírito Santo | 2017 | South American I |
| 2829 | MF170974 | Brazil - Espírito Santo | 2017 | South American I |
| M105 | MH018090 | Brazil - Minas Gerais | 2017 | South American I |
| M218 | MH018099 | Brazil - Bahia | 2017 | South American I |
| BeH655417 | JF912190 | Brazil - Roraima | 2002 | South American I |
| 2A | KM388817 | Venezuela | 2004 | South American I |
| 6A | KM388814 | Venezuela | 2005 | South American I |
| 10A | KM388816 | Venezuela | 2010 | South American I |
| 9A | KM388815 | Venezuela | 2007 | South American I |
| 8A | KM388818 | Venezuela | 2006 | South American I |
| 1 | MG969501 | Brazil - Minas Gerais | 2001 | South American I |
| BeAR646536 | JF912189 | Brazil - Rio Grande do Sul | 2001 | South American I |
| BeH622493 | JF912188 | Brazil - Goiás | 2000 | South American I |
| BeH622205 | JF912187 | Brazil - Goiás | 2000 | South American I |
| M225 | MH018100 | Brazil - Minas Gerais | 2003 | South American I |
| TVP11767 | HM582851 | Trinidad and Tobago | 2009 | South American I |
| M226 | MH018101 | Brazil - Minas Gerais | 2003 | South American I |
| Suriname2017 | MF347613 | Suriname | 2017 | South American I |
| BeAR378600 | JF912179 | Brazil - Goiás | 1980 | South American I |
| BeH463676 | JF912184 | Brazil - Pará | 1987 | South American I |
| BeH423602 | JF912183 | Brazil - Pará | 1984 | South American I |
| BeH394880 | JF912180 | Brazil - Pará | 1981 | South American I |
| BeAR513008 | JF912185 | Brazil - Mato Grosso do Sul | 1992 | South American I |
| BeH422973 | JF912182 | Brazil - Pará | 1984 | South American I |
| BeH526722 | JF912186 | Brazil - Minas Gerais | 1994 | South American I |
| Bolivia88 | MF004382 | Bolivia | 1999 | South American II |
| BeH413820 | JF912181 | Brazil - Rondônia | 1983 | South American II |
| JoseCachatra | KU978765 | Guinea-Bissau | 1965 | West African |
| Asibi | MF405338 | Ghana | 1927 | West African |
| M185D160 | KU978764 | Sudan | 1941 | West African |
| ArD181564 | JX898880 | Senegal | 2005 | West African |
| ArD181250 | JX898878 | Senegal | 2005 | West African |
| ArD181464 | JX898877 | Senegal | 2005 | West African |
| DakArAmt7 | JX898869 | Côte d'Ivoire | 1973 | West African |
| case1 | GQ379162 | Peru | 2007 | West African |
| ArD156468 | JX898876 | Senegal | 2001 | West African |
| case2 | GQ379163 | Peru | 2007 | West African |
| Ap7M | MF926243 | France | 2015 | West African |
| YFVEHI | MF289572 | Singapore | 2017 | West African |
| 17D | JX949181 | United States of America | 2012 | West African |
| ArD121040 | JX898870 | Senegal | 1996 | West African |
| HD117294 | JX898868 | Senegal | 1995 | West African |
| YFAVD279193F | DQ118157 | Spain | 2004 | West African |
| ArD149815 | JX898875 | Senegal | 2000 | West African |

| | | | | |
|---|---|---|---|---|
| ArD149194 | JX898874 | Senegal | 2000 | West African |
| ArD149214 | JX898873 | Senegal | 2000 | West African |
| ArD114972 | JX898872 | Senegal | 1995 | West African |
| ArD114896 | JX898871 | Senegal | 1995 | West African |
| BJ01 | KY495641 | China | 2016 | East African |
| CNYF01R | KX268355 | China | 2016 | East African |
| CIC4 | KX027336 | China | 2016 | East African |
| CIC2 | KX010995 | China | 2016 | East African |
| CNFY0321 | KY873607 | China | 2016 | East African |
| FJYF03 | KY587416 | China | 2016 | East African |
| CNYF01 | KU921608 | China | 2016 | East African |
| CIC3 | KX010996 | China | 2016 | East African |
| YFV003FIG | MK583147 | Brazil - São Paulo | 2018 | South American I |
| YFV004FIG | MK583148 | Brazil - São Paulo | 2018 | South American I |
| YFV005FIG | MK583149 | Brazil - São Paulo | 2018 | South American I |
| YFV007FIG | MK583150 | Brazil - São Paulo | 2018 | South American I |
| YFV008FIG | MK583151 | Brazil - São Paulo | 2018 | South American I |
| YFV010FIG | MK583152 | Brazil - São Paulo | 2018 | South American I |
| YFV015FIG | MK583153 | Brazil - São Paulo | 2018 | South American I |
| YFV016FIG | MK583154 | Brazil - São Paulo | 2018 | South American I |
| YFV018FIG | MK583155 | Brazil - São Paulo | 2018 | South American I |
| YFV020FIG | MK583156 | Brazil - São Paulo | 2018 | South American I |
| YFV021FIG | MK583157 | Brazil - São Paulo | 2018 | South American I |
| YFV022FIG | MK583158 | Brazil - São Paulo | 2018 | South American I |
| YFV023FIG | MK583159 | Brazil - São Paulo | 2018 | South American I |
| YFV029FIG | MK583160 | Brazil - São Paulo | 2018 | South American I |
| YFV033FIG | MK583161 | Brazil - São Paulo | 2018 | South American I |
| YFV038FIG | MK583162 | Brazil - São Paulo | 2018 | South American I |
| YFV040FIG | MK583163 | Brazil - São Paulo | 2018 | South American I |
| YFV041FIG | MK583164 | Brazil - São Paulo | 2018 | South American I |
| YFV042FIG | MK583165 | Brazil - São Paulo | 2018 | South American I |
| YFV043FIG | MK583166 | Brazil - São Paulo | 2018 | South American I |
| YFV047FIG | MK583167 | Brazil - São Paulo | 2018 | South American I |
| YFV048FIG | MK583168 | Brazil - São Paulo | 2018 | South American I |
| YFV052FIG | MK583169 | Brazil - São Paulo | 2018 | South American I |
| YFV053FIG | MK583170 | Brazil - São Paulo | 2018 | South American I |
| YFV054FIG | MK583171 | Brazil - São Paulo | 2018 | South American I |
| YFV056FIG | MK583172 | Brazil - São Paulo | 2018 | South American I |
| YFV060FIG | MK583173 | Brazil - São Paulo | 2018 | South American I |
| YFV061FIG | MK583174 | Brazil - São Paulo | 2018 | South American I |
| YFV065FIG | MK583175 | Brazil - São Paulo | 2018 | South American I |
| YFV066FIG | MK583176 | Brazil - São Paulo | 2018 | South American I |
| YFV070FIG | MK583177 | Brazil - São Paulo | 2018 | South American I |
| YFV072FIG | MK583178 | Brazil - São Paulo | 2018 | South American I |
| YFV074FIG | MK583179 | Brazil - São Paulo | 2018 | South American I |
| YFV076FIG | MK583180 | Brazil - São Paulo | 2018 | South American I |

| | | | | |
|---|---|---|---|---|
| YFV080FIG | MK583181 | Brazil - São Paulo | 2018 | South American I |
| YFV081FIG | MK583182 | Brazil - São Paulo | 2018 | South American I |

**Supplementary Table 3.** Complete genomes sequences used in the phylogeographic and phylodynamics analysis.

| Strain name | GenBank number | Local of isolation (Brazilian state, City) | Date of isolation | Isolation source | Host | Outbreak clade |
|---|---|---|---|---|---|---|
| YFV112 | MF465805 | Minas Gerais, Januária | 17-Jan-17 | Urine | *Homo sapiens* | Clade I |
| ES505 | KY885001 | Espírito Santo, Domingos Martins | 22-Feb-17 | Serum | *Alouatta clamitans* | Clade I |
| PA196 | MF423374 | Espírito Santo, Domingos Martins | 23-Feb-17 | Mosquitoes pool | *Haemagogus janthinomys* | Clade I |
| PA193 | MF423373 | Espírito Santo, Domingos Martins | 21-Feb-17 | Mosquitoes pool | *Haemagogus leucocelaenus* | Clade I |
| H199 | MF434851 | Rio de Janeiro, Casimiro de Abreu | 25-Apr-17 | Serum | *Homo sapiens* | Clade I |
| ES504 | KY885000 | Espírito Santo, Domingos Martins | 20-Feb-17 | Serum | *Alouatta clamitans* | Clade I |
| 2829 | MF170974 | Espírito Santo, Cariacica | 29-Mar-17 | Serum | *Homo sapiens* | Clade I |
| M105 | MH018090 | Minas Gerais, Caratinga | 12-Jan-17 | Liver | *Homo sapiens* | Clade I |
| M218 | MH018099 | Bahia, Cordeiros | 10-Mar-17 | NA | Non-human primate | Clade I |
| M211 | MH018096 | Minas Gerais, Jose Raydan | 13-Jan-17 | Liver | *Alouatta sp.* | Clade I |
| M210 | MH018095 | Minas Gerais, Sabinopolis | 19-Jan-17 | Liver | *Callithrix sp.* | Clade I |
| M138 | MH018093 | Minas Gerais, Santa Barbara Leste | 20-Jan-17 | Serum | *Homo sapiens* | Clade I |
| M123 | MH018092 | Minas Gerais, Itambacuri | 27-Jan-17 | Serum | *Homo sapiens* | Clade I |
| M68 | MH018080 | Minas Gerais, Ladainha | 28-Jan-17 | Liver | *Homo sapiens* | Clade I |
| M58 | MH018079 | Minas Gerais, Itambacuri | 28-Jan-17 | Liver | *Homo sapiens* | Clade I |
| M51 | MH018078 | Minas Gerais, Novo Cruzeiro | 18-Jan-17 | Liver | *Homo sapiens* | Clade I |
| 3495 | MF170981 | Espírito Santo, Santa Leopoldina | 05-Apr-17 | Serum | *Homo sapiens* | Clade I |
| 2563 | MF170980 | Espírito Santo, Alfredo Chaves | 23-Mar-17 | Serum | *Homo sapiens* | Clade I |
| 3472 | MF170979 | Espírito Santo, Santa Maria de Jetiba | 06-Apr-17 | Serum | *Homo sapiens* | Clade I |
| 2841 | MF170978 | Espírito Santo, Santa Maria de Jetiba | 28-Mar-17 | Serum | *Homo sapiens* | Clade I |
| 1122 | MF170977 | Espírito Santo, Conceicao do Castelo | 10-Feb-17 | Serum | *Homo sapiens* | Clade I |
| 282 | MF170976 | Espírito Santo, Laranja da Terra | 25-Jan-17 | Serum | *Homo sapiens* | Clade I |
| 1555 | MF170975 | Espírito Santo, Brejetuba | 03-Mar-17 | Serum | *Homo sapiens* | Clade I |
| 3919 | MF170973 | Brazil: Espírito Santo, Domingos Martins | 10-Apr-17 | Serum | *Homo sapiens* | Clade I |
| 2992 | MF170972 | Espírito Santo, Cariacica | 31-Mar-17 | Serum | *Homo sapiens* | Clade I |
| es2487 | MF170970 | Espírito Santo, Domingos Martins | 21-Mar-17 | Serum | *Homo sapiens* | Clade I |
| rj1930 | MF170969 | Espírito Santo, Casimiro de Abreu | 18-Mar-17 | Serum | *Homo sapiens* | Clade I |

| | | | | | | |
|---|---|---|---|---|---|---|
| 3925 | MF170968 | Espírito Santo, Marechal Floriano | 13-Apr-17 | Serum | *Homo sapiens* | Clade I |
| RJ104 | MF538786 | Rio de Janeiro, Guapimirim | 05-Jun-17 | Serum | Marmoset | Clade I |
| RJ97 | MF538785 | Rio de Janeiro, Petropolis | 21-Apr-17 | Serum | Marmoset | Clade I |
| H196 | MF538784 | Rio de Janeiro, Porciuncula | 26-Feb-17 | Serum | *Homo sapiens* | Clade I |
| H191 | MF538783 | Rio de Janeiro, Casimiro de Abreu | 18-Mar-17 | Serum | *Homo sapiens* | Clade I |
| H190 | MF538782 | Rio de Janeiro, São Fidelis | 16-Mar-17 | Serum | *Homo sapiens* | Clade I |
| RJ96 | MF423378 | Rio de Janeiro, Macaé | 19-Apr-17 | Serum | *Alouatta guariba clamitans* | Clade I |
| RJ95 | MF423377 | Rio de Janeiro, Carmo | 19-Apr-17 | Serum | *Alouatta guariba clamitans* | Clade I |
| RJ94 | MF423376 | Rio de Janeiro, Macaé | 13-Apr-17 | Serum | *Alouatta guariba clamitans* | Clade I |
| RJ87 | MF423375 | Rio de Janeiro, Macaé | 04-Apr-17 | Serum | *Alouatta guariba clamitans* | Clade I |
| YFV048FIG | MK583168 | São Paulo, São Paulo | 11-Feb-18 | Liver | *Homo sapiens* | Clade I |
| YFV016FIG | MK583154 | São Paulo, São Paulo | 21-Jan-18 | Liver | *Homo sapiens* | Clade I |
| m6590PNH | MF170971 | Minas Gerais, São Roque de Minas | 30-Jan-17 | Serum | New World monkey | Clade II |
| YFV003FIG | MK583147 | São Paulo, Mairiporã | 12-Jan-18 | Liver | *Homo sapiens* | Clade II |
| YFV004FIG | MK583148 | São Paulo, Mairiporã | 13-Jan-18 | Liver | *Homo sapiens* | Clade II |
| YFV005FIG | MK583149 | São Paulo, São Paulo | 16-Jan-18 | Liver | *Homo sapiens* | Clade II |
| YFV007FIG | MK583150 | São Paulo, Itaquaquecetuba | 15-Jan-18 | Liver | *Homo sapiens* | Clade II |
| YFV008FIG | MK583151 | São Paulo, Mairiporã | 15-Jan-18 | Liver | *Homo sapiens* | Clade II |
| YFV010FIG | MK583152 | São Paulo, Ibiúna | 16-Jan-17 | Liver | *Homo sapiens* | Clade II |
| YFV015FIG | MK583153 | São Paulo, Mairiporã | 20-Jan-18 | Liver | *Homo sapiens* | Clade II |
| YFV018FIG | MK583155 | São Paulo, Mairiporã | 22-Jan-18 | Liver | *Homo sapiens* | Clade II |
| YFV020FIG | MK583156 | São Paulo, Atibaia | 23-Jan-18 | Liver | *Homo sapiens* | Clade II |
| YFV021FIG | MK583157 | São Paulo, Guarulhos | 23-Jan-18 | Liver | *Homo sapiens* | Clade II |
| YFV022FIG | MK583158 | São Paulo, Mairiporã | 23-Jan-18 | Liver | *Homo sapiens* | Clade II |
| YFV023FIG | MK583159 | São Paulo, Atibaia | 24-Jan-18 | Liver | *Homo sapiens* | Clade II |
| YFV029FIG | MK583160 | São Paulo, Cotia | 29-Jan-18 | Liver | *Homo sapiens* | Clade II |
| YFV033FIG | MK583161 | São Paulo, Itaquaquecetuba | 24-Jan-18 | Liver | *Homo sapiens* | Clade II |
| YFV038FIG | MK583162 | São Paulo, São Paulo | 31-Jan-18 | Liver | *Homo sapiens* | Clade II |

| | | | | | | |
|---|---|---|---|---|---|---|
| YFV040FIG | MK583163 | São Paulo, São Paulo | 02-Feb-18 | Liver | *Homo sapiens* | Clade II |
| YFV041FIG | MK583164 | São Paulo, Cotia | 03-Feb-18 | Liver | *Homo sapiens* | Clade II |
| YFV042FIG | MK583165 | São Paulo, Mairiporã | 04-Feb-18 | Liver | *Homo sapiens* | Clade II |
| YFV043FIG | MK583166 | São Paulo, São Paulo | 06-Feb-18 | Liver | *Homo sapiens* | Clade II |
| YFV047FIG | MK583167 | São Paulo, Guarulhos | 11-Feb-18 | Liver | *Homo sapiens* | Clade II |
| YFV052FIG | MK583169 | São Paulo, Guarulhos | 18-Feb-18 | Liver | *Homo sapiens* | Clade II |
| YFV053FIG | MK583170 | São Paulo, Guarulhos | 18-Feb-18 | Liver | *Homo sapiens* | Clade II |
| YFV054FIG | MK583171 | São Paulo, Guarulhos | 18-Feb-18 | Liver | *Homo sapiens* | Clade II |
| YFV056FIG | MK583172 | São Paulo, Guarulhos | 19-Feb-18 | Liver | *Homo sapiens* | Clade II |
| YFV060FIG | MK583173 | São Paulo, Guarulhos | 23-Feb-18 | Liver | *Homo sapiens* | Clade II |
| YFV061FIG | MK583174 | São Paulo, Arujá | 24-Feb-18 | Liver | *Homo sapiens* | Clade II |
| YFV065FIG | MK583175 | São Paulo, Itaquaquecetuba | 27-Feb-18 | Liver | *Homo sapiens* | Clade II |
| YFV066FIG | MK583176 | São Paulo, Piedade | 27-Feb-18 | Liver | *Homo sapiens* | Clade II |
| YFV070FIG | MK583177 | São Paulo, Guarulhos | 04-Mar-18 | Liver | *Homo sapiens* | Clade II |
| YFV072FIG | MK583178 | São Paulo, Itariri | 10-Mar-18 | Liver | *Homo sapiens* | Clade II |
| YFV074FIG | MK583179 | São Paulo, Ibiúna | 11-Mar-18 | Liver | *Homo sapiens* | Clade II |
| YFV076FIG | MK583180 | São Paulo, Guarulhos | 14-Mar-18 | Liver | *Homo sapiens* | Clade II |
| YFV080FIG | MK583181 | São Paulo, São Lourenço da Serra | 27-Mar-18 | Liver | *Homo sapiens* | Clade II |
| YFV081FIG | MK583182 | São Paulo, Guarulhos | 14-Mar-18 | Liver | *Homo sapiens* | Clade II |

**Supplementary Table 4.** Comparison among continuous spatial diffusion models for Brazilian sequences from 2017-2018.

| | **Brazilian outbreak sequences (2017-2018)** | | |
|---|---|---|---|
| **Spatial diffusion model** | **Continuous** | | |
| **Demographic model** | **PS** | **SS** | **SS Log BF** |
| Constant | -16570.65 | -16570.97 | 0.0 |
| Exponential | -16565.27 | -16565.12 | 5.85 |
| **Logistic** | **-16562.77** | **-16562.85** | **8.12** |

PS: Path sampling; SS: Stepping stone.

**Supplementary Table 5.** Comparison among continuous diffusion models for Brazilian sequences from 2017-2018.

| **Continuous diffusion models** | **PS** | **SS** | **Marginal LnL (stdev)** | **Coefficient of variation (95% HPD)** | **Correlation (95% HPD)** | **SS Log** BF | **Dispersal rate (km/day) (95% HPD)** |
|---|---|---|---|---|---|---|---|
| BD | -16562.77 | -16562.85 | -16286.3 (10.52) | 1.11 (0.6, 1.66) | 0.56 (0.38, 0.74) | 0.0 | 4.00 (2.7, 5.33) |
| Cauchy RRW model | -16551.53 | -16551.43 | -16231.44 (11.14) | 0.77 (0.37, 1.21) | 0.35 (0.07, 0.62) | 11.42 | 3.21 (2.2, 4.29) |
| Gamma RRW model | -16551.58 | -16551.94 | -16234.35 (11.81) | 0.79 (0.39, 1.2) | 0.26 (-0.02, 0.55) | 10.91 | 3.12 (2.13, 4.2) |
| **Lognormal RRW model** | **-16546.49** | **-16546.95** | **-16232.95 (11.41)** | **0.84 (0.44, 1.27)** | **0.36 (0.09, 0.62)** | **15.9** | **3.3 (2.25, 4.37)** |

BD: Brownian diffusion process; RRWs: Relaxed random walks; PS: Path sampling; SS: Stepping stone. The log Bayes factor (BF) is the difference of the log ML between alternative (H1) and null (H0) models. Log BFs >3 indicates that model H1 is more strongly supported by the data than model H0. 0 < BF < 2 is not worth more than a bare mention, 2 < BF < 6 is positive evidence, 6 < BF < 10 is strong support, and BF > 10 is decisive.

**Supplementary Table 6.** Total number of sampled cases (n = 81) and qRT-PCR positive YFV (n = 67) in the sampled cities of São Paulo, Brazil (2017-2018).

| | | **YFV cases (n = 67)** | |
|---|---|---|---|
| **City** | **Cases (n = 81)** | **n** | **%** |
| Arujá | 1 | 1 | 100.0 |
| Atibaia | 2 | 2 | 100.0 |
| Carapicuíba | 1 | 1 | 100.0 |
| Cotia | 4 | 4 | 100.0 |
| Embu das Artes | 1 | 1 | 100.0 |
| Ferraz de Vasconcelos | 1 | 0 | 0.0 |
| Franco da Rocha | 2 | 1 | 50.0 |
| Guarulhos | 17 | 16 | 94.1 |
| Ibiúna | 3 | 3 | 100.0 |
| Itaquaquecetuba | 4 | 4 | 100.0 |
| Itariri | 1 | 1 | 100.0 |
| Mairiporã | 12 | 11 | 91.7 |
| Peruíbe | 1 | 1 | 100.0 |
| Piedade | 1 | 1 | 100.0 |
| São Bento do Sapucaí | 1 | 1 | 100.0 |
| São Lourenço da Serra | 1 | 1 | 100.0 |
| São Paulo | 28 | 18 | 64.3 |
| TOTAL | 81 | 67 | 82.7 |
